# Tailoring interval training in stroke rehabilitation: The role of peak velocity

**DOI:** 10.46439/rehabilitation.6.035

**Published:** 2025

**Authors:** Sangeetha Madhavan, Brice Cleland, Aditi Doshi, Cemal Ozemek

**Affiliations:** 1 Brain Plasticity Laboratory, Department of Physical Therapy, College of Applied Health Sciences, University of Illinois Chicago, Chicago, IL, USA; 2 Graduate Program in Neuroscience, University of Illinois Chicago, Chicago, IL, USA; 3 Department of Physical Therapy, College of Applied Health Sciences, University of Illinois Chicago, Chicago, IL, USA

**Keywords:** Stroke, Treadmill training, High intensity, Walking

## Abstract

Stroke rehabilitation demands innovative approaches to address the diverse physical limitations and functional goals of stroke survivors. Traditional interval training paradigms, such as high-intensity interval training (HIIT) and sprint interval training (SIT), often fall short of meeting the specific needs of this population due to their reliance on sustained or maximal efforts. Peak velocity interval training (PVIT) on the other hand, is an adaptable protocol tailored to optimize gait recovery in stroke survivors. PVIT emphasizes achieving individualized peak walking velocities during short, structured intervals, ensuring safe and effective intensity modulation across a range of functional abilities. This approach incorporates a 2-minute ramp-up phase to facilitate a gradual and safe progression to peak velocity, followed by a brief peak-effort phase and controlled recovery. The protocol is designed to accommodate stroke-specific challenges, including cardiovascular limitations, neuromuscular impairments, and fatigue. PVIT not only prioritizes safety and feasibility but also aligns with the principles of task-specific rehabilitation, focusing on improving walking speed, endurance, and overground functionality. In this manuscript, we present the rationale, structure, and implementation of the PVIT protocol, highlighting its distinctions from HIIT and SIT. We detail how PVIT addresses the physiological and practical limitations of stroke survivors while optimizing training outcomes. This work aims to redefine interval training for stroke gait training, offering practitioners a safe, scalable, and evidence-based strategy to enhance recovery outcomes. Preliminary data supporting the feasibility and efficacy of PVIT are also discussed to contextualize its application in clinical practice.

## Introduction

Stroke is a leading cause of death and disability worldwide [1]. Around 70% of individuals with stroke have persistent lower limb impairments [2,3], such as asymmetrical gait, reduced walking endurance, and slower walking speeds [4–6]. Typically, individuals post-stroke walk at speeds averaging 0.3 to 0.8 m/s, falling short of the 1.32 m/s needed for safe and independent ambulation [7,8]. These limitations lower quality of life and increase the risk of recurrent strokes, highlighting the need for post-stroke rehabilitation to improve gait speed and endurance [9–12].

Treadmill training is widely used in outpatient rehabilitation for stroke survivors due to its ability to provide a safe and controlled environment that facilitates task-specific practice [9,13,14]. Treadmill training effectively improves walking speed and endurance after a stroke [15]. However, these improvements have been modest and short-term [16]. Current clinical practice guidelines [9,17] advocate high-intensity training to maximize gait recovery after stroke [18]. High-intensity interval training (HIIT) has gained recognition as a particularly effective form of treadmill training, offering intermittent bursts of high-intensity walking alternated with recovery periods. This form of training has been shown to improve walking speed, endurance, and cardiovascular fitness, often outperforming moderate-intensity continuous training (MICT) in these areas [19–21].

Traditionally, accurately prescribing HIIT for an individual relies on metrics based on their measured maximal responses to a graded exercise test [20,22]. The well-studied 4×4 method consists of 4-minute high-intensity bouts at 70–90% of maximal heart rate (HR_max_) or 60–85% heart rate reserve (HRR) interspersed by 3-minute recovery periods at 40–50% HR_max_, performed 4 times. However, these recommendations may not suit stroke survivors, as nearly 70% stroke survivors often experience functional impairments that can affect their hemodynamic responses to exercise and their ability to put forth a true maximal effort [23–26]. Stroke-related motor impairments, such as decreased lower limb strength, poor coordination, and asymmetrical gait patterns, limit the ability to achieve walking speeds that correspond to objectively determined high-intensity efforts [4–6]. As a result, alternative approaches that are better suited to the unique needs of stroke survivors are increasingly being explored.

Velocity-based treadmill training is an alternative approach that shifts the focus from exercising at a percentage of maximal effort to treadmill belt velocity as the primary intensity measure. This approach emphasizes the participant’s peak achievable walking velocity during intervals, allowing for precise, individualized intensity adjustments that accommodate functional limitations. By prioritizing velocity as an intensity measure, these protocols align closely with the principles of task-specific rehabilitation, improving the relevance and efficacy of training for stroke survivors. Comparative studies within healthy populations demonstrated that velocity-based training protocols yielded superior gains in power and endurance compared to HR-based protocols [27]. Furthermore, PVIT prioritizes achieving each participant’s peak walking velocity during short, structured intervals, with careful modulation of rampup, peak, and recovery phases to ensure safety and feasibility. This flexible and individualized approach addresses the physiological and functional limitations of stroke survivors while maximizing gains in walking speed and endurance. It also aligns well with the emerging focus on individualized precision medicine for stroke rehabilitation [28,29]. Studies using protocols similar to PVIT in stroke have also shown promise in delivering substantial improvements in overground walking speed and endurance [20,30–32].

Accordingly, this paper outlines a step-by-step framework for the implementation of PVIT, addressing critical considerations for safety, monitoring, and progression. Preliminary descriptive data are included to contextualize the protocol and support its application in clinical practice.

## Training Overview and Rationale

These instructions are tailored for clinicians to implement in practice, ensuring both safety and effectiveness. This protocol has previously been validated by our group [32,33]. The PVIT protocol incorporates tailored interval treadmill walking to maximize gait recovery by emphasizing task-specific and individualized peak velocity efforts. Velocity, rather than heart rate (HR), is used to guide training, addressing the limitations in physiological responsiveness and capacity often seen in stroke survivors. The primary goal of PVIT is to continuously achieve increased maximum belt velocity in every session. Participants can typically complete multiple sessions over several weeks, with training tailored to their peak walking velocity as measured weekly using the 10-Meter Walk Test (10MWT).

## Setup and Equipment

To ensure safety and efficiency, the training setup includes a motorized treadmill with variable velocity settings and a safety stop feature to accommodate individualized training intensities. Participants are secured in an overhead safety harness without body-weight support to prevent falls during peak velocity walking; if an overhead harness is unavailable, a gait belt may be used as an alternative to ensure safety and stability. Handrails are available for support; however, participants are encouraged to minimize reliance to promote natural gait patterns. Continuous HR monitoring is conducted using a chest strap or wrist-based device, and blood pressure is measured before and after sessions, and as needed during training. Additional adjustments to treadmill velocity and harness fit are made at the start of each session to ensure both safety and comfort. The training area is well-lit, spacious, and equipped with easy access to emergency stop mechanisms and medical supplies.

## Session Components

Each session consists of three main components: warm-up, peak velocity intervals, and cool-down. Participants begin with a 5-minute warm-up at 50% of their peak weekly walking speed, as determined by the 10MWT. The peak velocity interval phase includes 20–40 minutes of velocity-based intervals interspersed with active recovery. Each interval consists of three phases:

1. Ramp-Up Phase: Gradual increase to peak velocity over 2 minutes.
2. Peak Velocity Phase: Walking at peak tolerable velocity for 10 seconds (progressing to 60 seconds over the course of the training intervention).
3. Ramp-Down Phase: Return to recovery velocity over 5 seconds.

**Figure 1.**
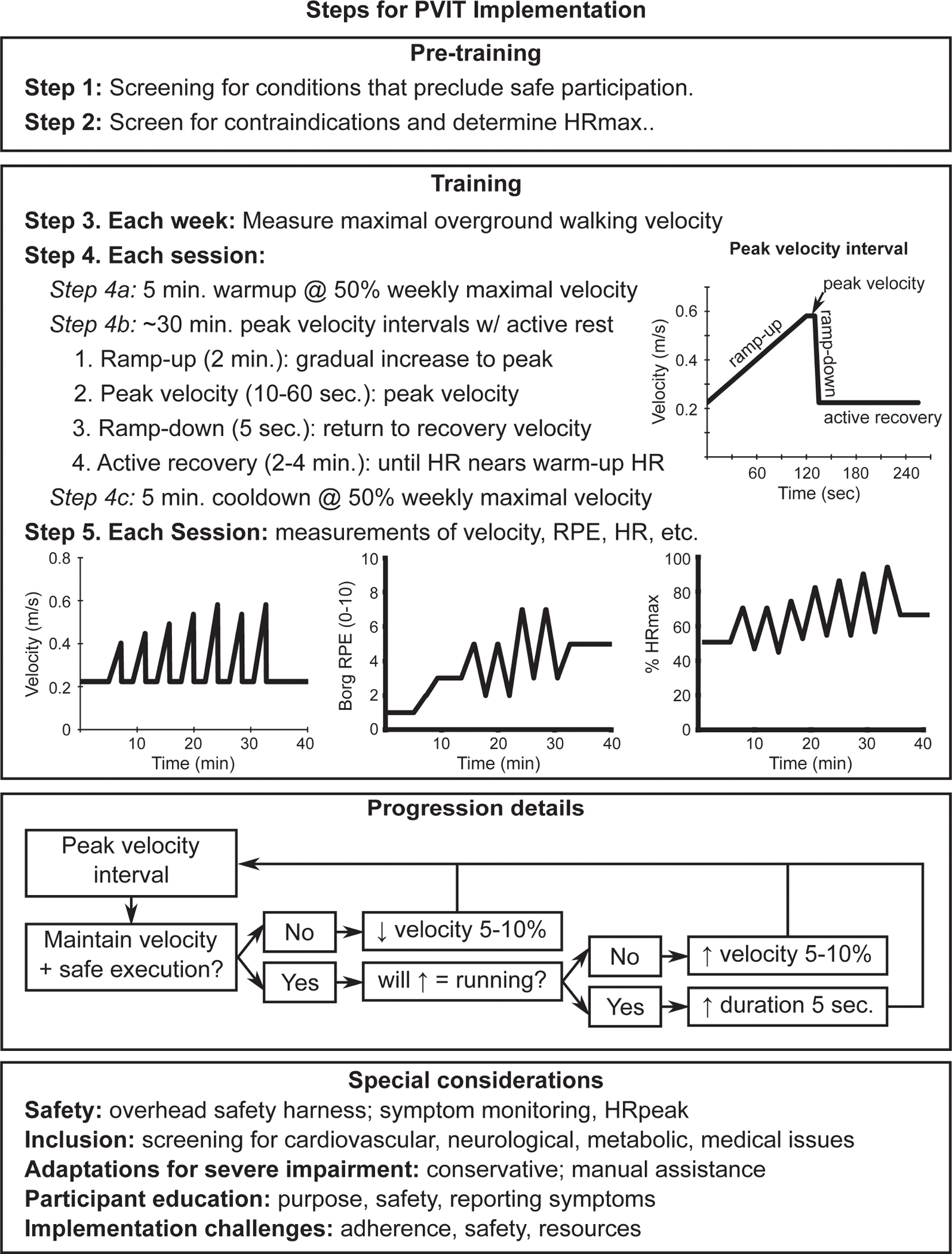
Schematic representation of the Progressive Velocity Interval Training (PVIT) protocol. The diagram outlines key implementation steps, including screening and weekly maximal velocity assessment. The structured training sessions consist of warm-up, peak velocity intervals (ramp-up, peak velocity, ramp-down, and active recovery), and cooldown, with adjustments based on participant response and safety considerations.

The active recovery period involves walking at warm-up velocity for at least 2 minutes or until the HR returns to within 5 beats per minute (bpm) of the warm-up HR. Session durations are adjusted based on participant tolerance, lasting between 20 and 40 minutes, including rest and pauses as needed. After completing the intervals, a 5-minute cool-down at 50% of peak weekly walking speed is performed to ensure safe recovery. This protocol is summarized in Figure 1.

## Progression Recommendations

Progression in PVIT should be tailored to each participant’s tolerance and performance. Training intensity can be adjusted by modifying treadmill velocity or the duration of intervals. Initial baseline testing, such as the 10MWT, is used to determine the participant’s peak walking speed. The obtained velocity will then be decreased by 50% and used for the 5-minute warm-up phase on the treadmill. After the warm-up, the first velocity-based training interval is initiated. During a period of ~2 minutes, the belt velocity will be increased, within the participant’s tolerance, to the highest velocity at which the participant can walk safely without stumbling. If the participant maintained the velocity and felt safe during the peak velocity achieved at the end of the first-training interval, the velocity will then be increased by 5– 10% during the next interval. During any fast-walking phase, if the participant is unable to maintain the required velocity and feels unsafe, the velocity will then be reduced by 5–10% for the next interval.

The duration of high-velocity intervals can also be gradually extended from 10 seconds to 60 seconds as tolerated. If signs of excessive fatigue or instability appear, adjustments should be made by reducing the treadmill velocity or interval duration by 5–10%. Regularly recording metrics such as peak HR, interval duration, and Borg RPE scores after each session ensures a comprehensive and individualized approach to progression.

Transition to running gait is monitored and avoided by capping velocity and instead increasing interval duration. However, if the clinician wishes to encourage running, this should be a collaborative decision made between the participant and clinician, taking into account the participant’s comfort, safety, and readiness. It is important to note that this protocol does not specifically provide guidance on transitioning to running, as there is limited research on this topic in stroke rehabilitation. For faster walkers, adjustments in velocity—whether increases or decreases—are kept to less than 10% to further reduce the risk of unintentional transition to running.

## Safety and Monitoring

Safety protocols are integral to the implementation of PVIT. An overhead safety harness is used without body-weight support to prevent falls, and participants have access to emergency stop mechanisms at all times. Handrails are available but minimal reliance is encouraged to promote natural gait patterns. Warm-up and cool-down periods should be extended for individuals with conditions such as hypertension or known angina. Real-time monitoring should be conducted during each session to ensure safety and efficacy. Continuous HR monitoring using a chest strap or wrist-based device is essential. Training is halted if the participant’s heart rate exceeds their peak HR (HRpeak) achieved during a graded exercise test or fails to return to the warm-up level within 4 minutes of recovery walking. This approach was adapted from our previous study; however, clinical judgment may be used to continue the training session in the absence of symptoms or inappropriate hemodynamic responses. Training is halted if the participant’s HR exceeds their HR_peak_ achieved during a graded exercise test or fails to return to the warm-up level within 4 minutes of recovery walking. This approach was adapted from our previous study; however, clinical judgment may be used to continue the training session in the absence of symptoms or inappropriate hemodynamic responses.

Participants are instructed to stand or sit as needed until HR recovery. The training intensity should be decreased if a participant reaches a HR previously determined to be associated with ischemia, arrhythmias or other symptoms. Blood pressure is measured before, during, and after training, and a hypertensive response, defined as systolic blood pressure greater than 200 mmHg or diastolic pressure exceeding 110 mmHg, is a clear indication to discontinue the session. The Borg RPE scale is used to track subjective exertion levels, and observers monitor for symptoms such as dizziness, chest pain, severe fatigue, or dyspnea. Immediate cessation of training is required if these symptoms occur. Observers also validate participant responses, particularly in cases where subjective reporting may be unreliable.

## Data Collection

Clinicians should record key metrics during each session to monitor progress and ensure safety. These include: HR at key phases (warm-up, intervals, recovery, and cool-down), Borg RPE scores, and treadmill metrics such as velocity, interval duration, total walking time, and distance covered. This data supports individualized progression and will enable evaluation of protocol efficacy.

## Special Considerations

### Inclusion and safety criteria

Prior to initiating training, participants must undergo thorough medical and functional evaluations to identify any conditions that might preclude safe participation. Key contraindications include:

1. Cardiovascular Conditions: Unstable angina, uncontrolled arrhythmias, or severe hypertension (resting blood pressure > 180/110 mmHg) are absolute contraindications.
2. Neurological Factors: Severe spasticity, balance impairments, or cognitive deficits that compromise the ability to follow instructions.
3. Metabolic Conditions: Poorly controlled diabetes (e.g., hypoglycemia or hyperglycemia with symptoms), severe autonomic dysfunction, or orthostatic hypotension.
4. Recent Medical Events: Stroke within the past three weeks, or recent cardiovascular interventions (e.g., myocardial infarction within four weeks or percutaneous coronary intervention within three weeks).

To mitigate risks, pre-training assessments should include cardiovascular screening, resting HR and blood pressure measurements, and evaluations for co-morbidities such as diabetes or renal dysfunction. Participants with relative contraindications may still participate with appropriate modifications and medical clearance.

### Adaptations for severely impaired participants

For participants with limited initial walking capacity, treadmill velocity and interval duration are adjusted conservatively. Manual assistance may be provided as necessary, with the goal of fostering independence over time.

### Participant education

Clinicians should educate participants about the purpose of the protocol, safety measures, and the importance of reporting symptoms such as fatigue, discomfort, or dizziness during training.

### Implementation challenges and considerations

We recognize that PVIT may require adaptations depending on setting and available resources. Key implementation barriers include the need for continuous supervision, access to safe treadmill systems with fall protection, and clinician familiarity with speed-based progression. However, unlike some robotic or HR-max-based systems, PVIT emphasizes practicality. It can be delivered using standard treadmills and safety harnesses or gait belts, without the need for advanced cardiovascular equipment. This enhances accessibility in typical outpatient rehabilitation environments. Clinicians may face challenges such as variations in participant adherence, safety concerns, or resource limitations (e.g., access to treadmills with appropriate safety features). Addressing these challenges requires adequate staff training, patient education, and regular protocol review. Ongoing clinician training and patient-tailored modifications are recommended to overcome these barriers.

## Example Participant Progressions in PVIT

**Figure 2.**
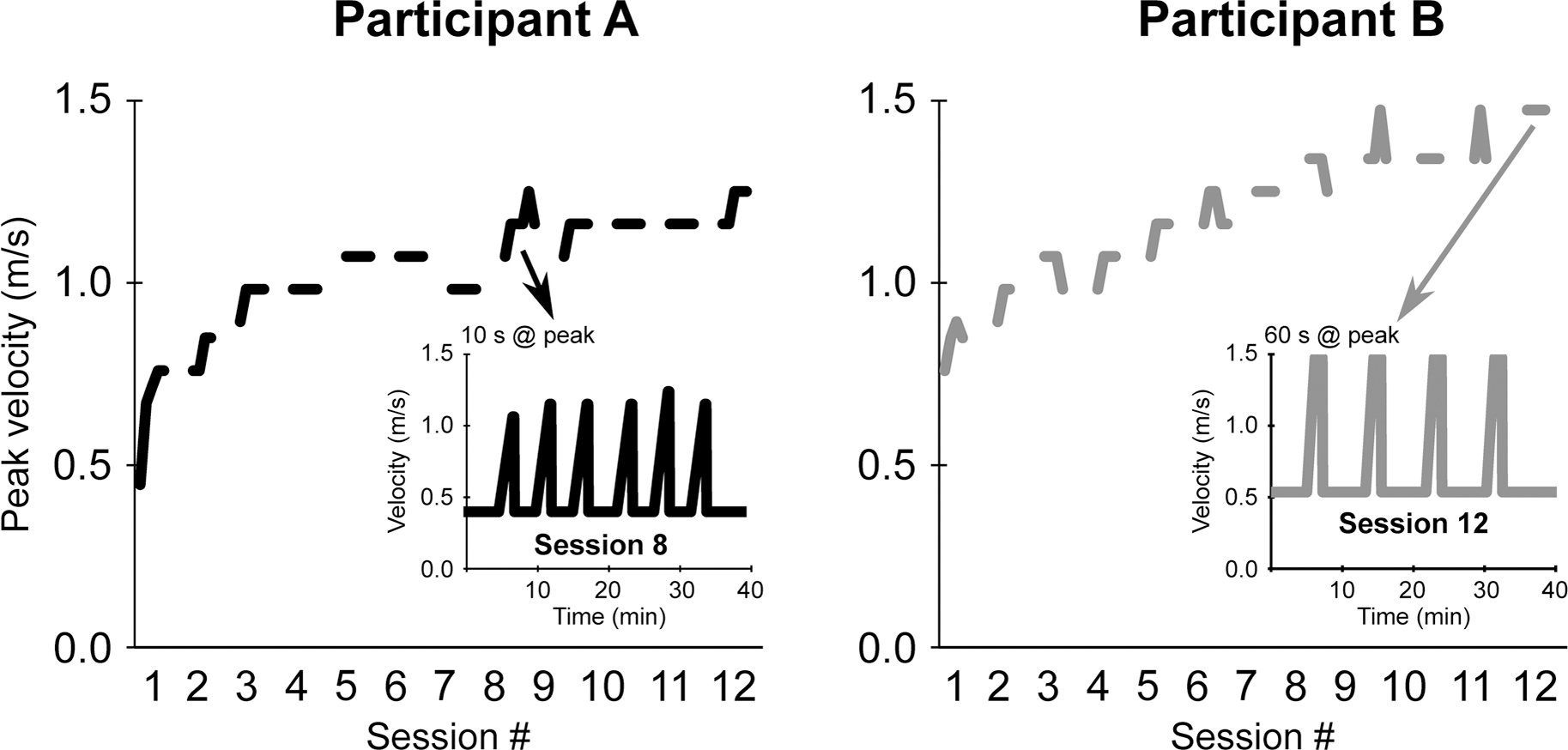
Velocity progression of participants A and B across training sessions. The main figure shows velocity (m/s) is plotted multiple sessions. The smaller inset shows the progression of peak velocity across a single session, highlighting individual adaptation including changes in peak velocity duration (10s vs. 60s) between different participants.

As part of a prior study investigating the effects of PVIT in individuals post-stroke, comprehensive data were collected to assess participant progression and the protocol’s impact on functional outcomes [32]. These data include detailed session-by-session treadmill performance metrics and pre- and post-intervention overground walking assessments. To illustrate the practical application and variability of the PVIT protocol, we present selected examples of two participants who demonstrated distinct response trajectories, emphasizing the adaptability and individualized nature of the intervention (Figure 2). Below, we detail their demographic characteristics, treadmill velocity improvements, and changes in overground walking speed to highlight key aspects of the protocol’s implementation and outcomes.

### Participant A

A 62-year-old male, three years post-stroke, with left hemisphere involvement and moderate gait impairment (baseline comfortable walking speed: 0.68 m/s (2.45 km/h); fast walking speed: 0.89 m/s (3.20 km/h)). Participant A demonstrated a steady and consistent progression in peak treadmill velocity throughout the 12-session intervention. Starting with a baseline peak treadmill velocity of 0.76 m/s (2.74 km/h), they exhibited modest gains in the first four sessions, reaching 1.0 m/s (3.60 km/h). By session nine, their peak velocity increased to 1.1 m/s, culminating in a final session velocity of 1.25 m/s (4.50 km/h). Additionally, their high-velocity interval durations improved from 10 seconds in the initial sessions to 45 seconds by the end of the protocol. HR responses supported their progressive improvements in performance. While their HR during peak intervals initially reached 61% of their age-predicted HRmax, it gradually stabilized at approximately 65% HRmax as they adapted to the protocol. Simultaneously, their RPE during peak intervals reached a score of 10 (maximal exertion) but decreased to 7 (somewhat severe) as they grew more comfortable sustaining higher velocities. These trends reflected both physiological and perceptual adaptations over time. Overground walking assessments showed improvements. Comfortable walking speed increased from 0.68 m/s (2.45 km/h) to 0.82 m/s (2.95 km/h), and fast walking speed rose from 0.89 m/s (3.20 km/h) to 1.1 m/s (3.96 km/h). These gains reflect both the transferability of treadmill-based training to overground walking and the potential for the PVIT protocol to enhance functional ambulation.

### Participant B

A 58-year-old female, six years post-stroke, with right hemisphere involvement and mild gait impairment (baseline comfortable walking speed: 0.82 m/s (2.95 km/h); fast walking speed: 1.02 m/s (3.67 km/h)). Participant B displayed rapid initial improvements, starting at a baseline peak treadmill velocity of 0.9 m/s (3.24 km/h) and reaching 1.26 m/s (4.54 km/h) by session six. Their progression plateaued in the later weeks, stabilizing at a peak treadmill velocity of 1.48 m/s (5.33 km/h) by session 10. However, interval duration steadily increased from 15 seconds in the first session to 60 seconds by the end, demonstrating enhanced endurance and sustained effort. Initial HR_peak_ values reached 92% age-predicted HRmax but stayed at approximately 91% HRmax with improved endurance and efficiency. RPE scores mirrored these changes, with peak interval scores starting at 5 (severe) and decreasing to 3 (moderate). Overground walking velocities mirrored these trends. Comfortable walking speed improved from 0.82 m/s (2.95 km/h) to 0.94 m/s (3.38 km/h), while fast walking speed increased from 1.02 m/s (3.67 km/h) to 1.24 m/s (4.46 km/h). These results underscore the variability in participant responses and the protocol’s flexibility in accommodating diverse patterns of improvement.

## Interpretation of Progressions

The inclusion of participant case studies in this manuscript demonstrates the practical application and flexibility of the PVIT protocol for post-stroke gait rehabilitation, highlighting its capacity to accommodate varied participant characteristics and yield significant improvements in treadmill velocity and overground walking ability. These examples illustrate the individualized nature of the intervention, underscoring its potential to address diverse rehabilitation needs in stroke survivors and reinforcing the value of velocity-based metrics as a primary measure for tailoring training intensity. While the case studies offer valuable insights, they represent a small subset of participants and may not capture the full spectrum of responses to the protocol, highlighting the need for future research to validate these findings in larger, more diverse cohorts and to explore long-term outcomes. Moreover, future studies should investigate optimal progression strategies, the protocol’s impact on other functional domains such as balance and endurance, and its scalability across different rehabilitation settings, as well as consider the integration of complementary interventions to enhance recovery. The observed improvements in functional outcomes align with current evidence supporting high-intensity interval training in stroke rehabilitation and suggest that velocity-based approaches may offer a practical alternative to HR-based protocols, particularly for individuals with cardiovascular or autonomic dysfunction.

## Discussion

The PVIT protocol offers a structured and replicable approach to enhancing gait rehabilitation outcomes, providing clinicians with a framework that balances safety, feasibility, and task-specific intensity. PVIT focuses on achieving each participant’s individualized peak velocity during intervals, aligning with the foundational principles of HIIT and SIT by promoting intervals of challenging effort interspersed with recovery. However, PVIT stands out as a targeted adaptation specifically designed for stroke rehabilitation, addressing the distinct physiological and functional challenges faced by stroke survivors, such as reduced exercise tolerance, impaired motor control, and blunted heart rate responses. Unlike traditional HIIT, which emphasizes HR zones, or SIT, which demands all-out maximal effort, PVIT leverages peak achievable walking speed as a proxy for intensity. This may make it inherently more adaptable to the heterogeneity in stroke recovery. In our prior studies [32,33], we demonstrated that speed-based treadmill training is not only safe but results in significant gains in walking speed and endurance. PVIT builds upon this by incorporating flexible velocity-based progression with tailored interval timing—preserving the intensity principles of HIIT while addressing the neuromotor and cardiorespiratory constraints often seen in stroke survivors.

Although walking is used as the primary example in this protocol, PVIT is inherently adaptable and can be applied to other activities depending on the patient’s capacity and rehabilitation goals. For instance, PVIT could be implemented in cycling, rowing, or even upper-limb-focused tasks for patients with significant lower-limb impairments. This adaptability makes the protocol relevant not only for stroke survivors but also for other populations with specific physical or neurological limitations, such as individuals recovering from traumatic brain injury, spinal cord injury, or those with degenerative conditions like Parkinson’s disease and multiple sclerosis.

Furthermore, integrating subjective measures such as perceived exertion alongside objective metrics like HR allows for a more nuanced and precise assessment of exercise intensity. This dual approach is particularly beneficial for populations with blunted HR responses, where HR-based prescriptions may not reliably reflect true effort or capacity. PVIT’s reliance on speed or velocity as an intensity measure ensures that clinicians can accurately and safely tailor rehabilitation interventions even for those with cardiovascular or autonomic dysfunction. This manuscript does not include a control group or long-term outcomes, its primary aim is to present a detailed and replicable protocol; future studies should incorporate controlled comparisons and follow-up assessments to evaluate both the efficacy and durability of the PVIT intervention.

## Conclusions

PVIT represents a practical evolution of interval training paradigms, combining the strengths of HIIT and SIT while introducing a task-specific and individualized focus that is particularly relevant for stroke survivors. Its adaptability across activities and populations further enhances its potential impact, making PVIT a valuable addition to the toolbox of rehabilitation strategies for clinicians worldwide. By adhering to core neuroplasticity principles and customizing the protocol to meet individual needs, clinicians can maximize the safety, effectiveness, and scalability of this approach in a variety of clinical settings.

## Abbreviations:

HIIT: High-Intensity Interval Training
HR: Heart Rate
HRmax: Maximal Heart Rate
HRR: Heart Rate Reserve
MICT: Moderate-Intensity Continuous Training
PVIT: Peak Velocity Interval Training
SIT: Sprint Interval Training
